# Identification of cucumber circular RNAs responsive to salt stress

**DOI:** 10.1186/s12870-019-1712-3

**Published:** 2019-04-27

**Authors:** Yong-Xing Zhu, Jian-Hua Jia, Lei Yang, Yu-Chen Xia, Hui-Li Zhang, Jin-Bu Jia, Ran Zhou, Pei-Yao Nie, Jun-Liang Yin, Dong-Fang Ma, Le-Cheng Liu

**Affiliations:** 1 Hubei Key Laboratory of Waterlogging Disaster and Agricultural Use of Wetland/College of Horticulture and Gardening/College of Agriculture, Yangtze University, Jingzhou, 434000 Hubei China; 2 College of Horticulture, Northwest A&F University, Yangling, 712100 Shaanxi China; 3 Department of Biology, Southern University of Science and Technology, Shenzhen, 518055 Guangdong China; 4 Biomarker Technologies, Beijing, 101300 China

**Keywords:** *Cucumis sativus*, Cucumber circRNAs, Salt stress, Parent genes, GO enrichment

## Abstract

**Background:**

Circular RNAs (circRNAs) are 3′-5′ head-to-tail covalently closed non-coding RNA that have been proved to play essential roles in many cellular and developmental processes. However, no information relate to cucumber circRNAs is available currently, especially under salt stress condition.

**Results:**

In this study, we sequenced circRNAs in cucumber and a total of 2787 were identified, with 1934 in root and 44 in leaf being differentially regulated under salt stress. Characteristics analysis of these circRNAs revealed following features: most of them are exon circRNAs (79.51%) and they prefer to arise from middle exon(s) of parent genes (2035/2516); moreover, most of circularization events (88.3%) use non-canonical-GT/AG splicing signals; last but not least, pairing-driven circularization is not the major way to generate cucumber circRNAs since very few circRNAs (18) contain sufficient flanking complementary sequences. Annotation and enrichment analysis of both parental genes and target mRNAs were launched to uncover the functions of differentially expressed circRNAs induced by salt stress. The results showed that circRNAs may be paly roles in salt stress response by mediating transcription, signal transcription, cell cycle, metabolism adaptation, and ion homeostasis related pathways. Moreover, circRNAs may function to regulate proline metabolisms through regulating associated biosynthesis and degradation genes.

**Conclusions:**

The present study identified large number of cucumber circRNAs and function annotation revealed their possible biological roles in response to salt stress. Our findings will lay a solid foundation for further structure and function studies of cucumber circRNAs.

**Electronic supplementary material:**

The online version of this article (10.1186/s12870-019-1712-3) contains supplementary material, which is available to authorized users.

## Background

Circular RNAs (circRNAs) have recently emerged as a new form of non-coding RNA in a wide variety of organisms [1]. Different from the traditional linear RNAs, they are special endogenous RNA molecules that are predominantly formed by 3′-5′ head-to-tail ligation in a backsplicing reaction. Although observed for decades in eukaryotic cells, circRNAs had been perceived as splicing errors in the past. Recently, knowledge of circRNAs has changed substantially. Thanks to the technological breakthroughs in high-throughput sequencing and high-efficiency bioinformatics analyzing strategies, a vast number of circRNAs have been identified in mice, humans, Archaea, and *Caenorhabditis elegans*, etc. [2]. Characterization analyses indicate that circRNAs can arise from exons (exonic circRNA), introns (intronic circRNA), and intergenic region and their expression patterns are often cell, tissue and developmental stage-specific [3]. Furthermore, evidence showed that circRNAs are much more stable than traditional linear RNAs and therefore might be involved in biological processes through a distinct way [3].

In recent years, the biological importance of circRNAs in transcriptional and post-transcriptional regulation have been increasingly illuminated. One of the most studied functions of circRNAs in animals is that they can function as microRNA (miRNA) sponge. For example, Zheng, et al. [4] reported that human *circHIPK3* has 18 potential binding sites and can sponge to 9 miRNAs. Wang, et al. [5] attested that a heart-related circRNA (*HRCR*) acts as a ceRNA to sponge miR-223 and inhibit its activity. Another remarkable role of circRNAs is to regulate parental gene expression. For example, in human, some circRNAs have been reported to promote the expression of their parental genes in *cis* via specific RNA-RNA interactions [6]. Besides, some circRNAs are even able to regulate cell cycle progression or ageing by directly interacting with protein [7, 8]. Despite the relevant functional roles of several circRNAs have been revealed, the majority of them are still mysterious to us, especially their functions in biological processes and associated regulating pathways.

In contrast to animals, to date, plant circRNAs have not been comprehensively identified. Wang, et al. [9] firstly carried out circRNA research in plant and found clear evidence for RNA circles in Arabidopsis. Meanwhile, Ye, et al. [10] identified 12,037 and 6012 circRNAs in rice (*Oryza sativa*) and Arabidopsis through genome-wide level inspecting, which confirmed the widespread of circRNAs in both monocot and dicot plants. Later, circRNAs have been identified in tomato [1, 11], potato [12], kiwifruit [13], wheat [14], soybean [15], tea [16], barely [17], and maize [18]. It is worth noting that although circRNA-miRNA-mRNA network has been constructed in many of these studies in plant, unlike in animals, miRNA sponge roles of circRNAs in plants has not been proven yet. Until now, functional studies on circRNAs are limited, especially in plants. Some studies suggested that circRNAs can regulate gene expression at the transcriptional and/or posttranscriptional levels by interacting with miRNAs [6, 14]. A transgenic study revealed that overexpression of a circRNA construct could reduce the expression level of its parental gene in transgenic rice, suggesting that circRNAs can act as a negative regulator for their parental genes. Similarly, in tomato, Tan, et al. [11] overexpressed a circRNA derived from a key gene in carotenoid biosynthesis (*Phytoene Synthase 1*), resulting in a decreasement of *Phytoene Synthase 1* mRNA abundance, as well as the lycopene and β-carotene accumulation. More recently, Cheng, et al. [19] reported that over-expression of a lariat-derived circRNA, generated from the first intron of AT5G37720, alters expression of approximately 800 genes and influence development of Arabidopsis. Moreover, an increasing number of studies in plants has also proposed that the circRNAs play important roles in regulating the response to environmental stresses. In rice, 27 exonic circRNAs were found to be differentially expressed under phosphate-sufficient and -starvation conditions, suggesting that circRNAs play roles in stress-specific biological processes in plants [10]. Similarly, in wheat, kiwifruit, and potato, circRNAs have been found to be specific responded to plant dehydration and bacterial stresses [12–14]. In Arabidopsis, Pan, et al. [20] revealed that heat stress induced more circRNAs than in normal condition, and these circRNAs may participate in plant response to heat stress through the circRNA-mediated ceRNA networks. These observations confirmed that circRNAs involved in a wide range of biological processes in plants, including growth and development, abiotic and biotic stress response.

Several characteristics of animal circRNAs, such as conservation, tissue-specific expression profiles, and longer flanking introns, are shared by plant circRNAs. Some distinct features of plant circRNAs have also been reported. For instance, plant seems to possess much less repetitive elements and reverse complementary sequences in their flanking introns [10]. Recently, Ye, et al. [21] found that large number of rice circRNAs (92.7%) are flanked by diverse non-GT/AG splicing signals while most human exonic circRNAs are flanked by canonical GT/AG splicing signals. The first achievement towards understanding the molecular mechanisms of plant circRNAs has been obtained in Arabidopsis. Conn, et al. [22] demonstrated that Arabidopsis circRNA generated from the exon 6 of the SEPALLATA3 (SEP3) genes regulates the expression of its parental gene (SEP3) by binding strongly to its cognate DNA locus (forming an RNA:DNA hybrid or R-loop), revealing the ability of specific circRNAs to skew splicing preference and favour the cognate alternative splicing (AS) mRNA variant. Furthermore, in maize, Chen et al. [18] hypothesized that transposons may be involved in the formation of circRNAs and they may further modulate phenotypic variation. Taken together, these studies provide novel insight into the biological function and generation mechanism of circRNAs in plant. Genome-wide discovery and characterization of circRNAs should be performed in more plants, which could provide a basis for further studies of the evolution, biogenesis, and function of circRNAs.

Salt stress is a major abiotic stress that limits plant growth and development. Currently, more than 20% of the world’s agricultural irrigated land is adversely affected by levels of salt that could markedly decrease the harvested agriculture yield [23]. Cucumber is an important controlled-environment horticultural crop and is fairly sensitive to salinity [24, 25]. Previously, non-coding RNAs, such as microRNA, have been shown to  play critical roles in regulating gene expression in response to abiotic stress including salt stress in plant [26–28]. However, to our knowledge, there is still no evidence showing the role of circRNAs, a new type of non-coding RNAs, in the salt stress resistance of cucumber. Besides, although genome-wide identification of circRNAs has been performed in multiple plant species, the characteristics and functions of circRNAs are not clear in cucumber. Our study firstly focused on identification and characterization of circRNAs in cucumber, and uncovered a potential functional role of circRNAs in the regulation of salt stress response.

## Methods

### Plant materials and treatments

Cucumber (*Cucumis sativus* L. ‘JinYou 1’, Xintiandi Co., Yangling, Shannxi, China) seeds were rinsed thoroughly in distilled water and germinated on moist gauze in an incubator at 28 °C for 2 days. The germinated seeds were sown in quartz sands in a greenhouse with an average day/night (12 h/12 h) temperature of 28 °C/18 °C. The seedlings were transferred to 15-L plastic containers filled with 1/4 strength of Hoagland nutrient solution at two-leaf stage, and each container had 10 plants. Three days later, the strength of Hoagland solution was increased to 1/2. Seven days after transplanting, salt treatment was started by adding sodium chloride (NaCl 75 mM) to the nutrient solution. The pH of nutrient solution was adjusted to 6.0 using 0.2 M H_2_SO_4_ or 1 M KOH.

### Growth parameters and root morphology traits

Dry weight was determined by collecting shoots and roots after 2, 4, 6, 8, 10, 12, 14, 16, 18 days’ of treatment, washing with distilled water, dried in an oven at 75 °C for 72 h, and then weighted. Roots were collected and determined by scanning roots with the root scanner platform (Model MRS-9600TFU2L, Microtek, Shanghai, China). The scanned images were analyzed with a root image analysis software (MRS-9600TFU2L, Zhongjing Technology Co. Ltd., Hangzhou, China). Each treatment included three replications and was successively performed in 2 independent experiments.

### CircRNA-seq

After 3 days of treatment, the second fully expanded leaves and roots were collected from 12 independent plants of the CK and NaCl-treated seedlings with every 6 of them being mixed as one biological replicate for each treatment and then frozen in liquid nitrogen immediately and stored at − 80 °C. All the treatments had 2 independent experiment replicates. The total RNA extraction and quality confirmation were performed according to the methods reported by Yin, et al. [1]. Then, following the manufacturer’s procedure, 2 μg of total RNA from each sample was treated with Ribo-Zero rRNA Removal Kit (for plant, Epicentre, Madison, WI, USA) to obtain ribosomal RNA (rRNA)-depleted RNA, and was further treated with RNase R (Epicentre, Madison, WI, USA) to eliminate linear RNAs. The remaining RNAs were used to generate circRNA-seq libraries according to Yin, et al. [1]. Then the libraries were sequenced on Illumina HiSeq™ X-ten platform at Biomarker Technologies Co., Ltd. (Beijing, China), and 150 bp pair-end reads were generated. The raw sequencing data were deposited in the US National Center for Biotechnology Information (NCBI) Sequence Read Archive (https://www.ncbi.nlm.nih.gov/sra) with a Bioproject ID: PRJNA449130.

### Identification of circRNAs

These low quality reads, including that (1) unknown (N) bases are more than 5%, (2) contain adaptor sequences, and (3) contain low quality bases (Q ≤ 20) more than 50%, were removed. The remaining high-quality reads were aligned against the previously reported *C. sativus* reference genome v2 using BWA (v0.7.15, mem -T 19). The output SAM files were then inspected by CIRI2 (v2.05), the considerable reliability and sensitive tool, to identify circRNAs [29]. Unanchored scaffolds in cucumber genome were merged as ChrUn (detailed information can be found in Additional file 1: Table S1). CircRNA expression levels were normalized to be SRPBM (SRPBM = number of circular reads/number of mapped reads (units in billion)/read length).

### Annotation and GO enrichment of circRNAs

Databases including Nr (NCBI non-redundant protein sequences), GO (Gene Ontology), KEGG (Kyoto Encyclopedia of Genes and Genomes), KOG/COG (Clusters of Orthologous Groups of proteins), Swiss-Prot (A manually annotated and reviewed protein sequence database, https://www.uniprot.org/), and Pfam (Protein family) were used to perform gene function annotation. The identification of differentially expressed circRNAs were performed using R package EBSeq. The cut-off criterion were set as |log_2_(Fold change)| > 1 and false discovery rate (FDR) adjusted *p*-value ≤0.05. GO annotations of the parent genes of differentially expressed circRNAs were collected and used to perform GO enrichment by topGO with default parameters. Veen and Weblogo diagrams were draw using online tools (http://bioinformatics.psb.ugent.be/webtools/Venn/, http://weblogo.berkeley.edu/logo.cgi). Circos and Violin diagrams were draw using correspondence R packages.

### Prediction for circRNA-miRNA-mRNA relationships

To construct the circRNA-miRNA-mRNA network, we collected the mature sequences of cucumber miRNAs from the previously reported publications [30–35] (Additional file 2: Table S2). Then the miRNAs identical alignment regions in circRNAs were predicted by TargetFinder using default parameters [36]. Meanwhile, cucumber coding genes (mRNAs) targeted by collected miRNAs were predicted by searching the selected library [*Cucumis sativus* (cucumber), cds, Cucumber genome sequencing project, version 2] with online tool (http://plantgrn.noble.org/psRNATarget/) [1]. The circRNA-miRNA-mRNA regulating network was generated by Cytoscape (v3.6.0) [37]. The eggNOG class annotations of these miRNAs targeted mRNAs were collected and used to interpret the possible regulating roles of circRNAs.

### Validation of circRNAs

To further confirm the circRNAs identified from cucumber, total RNA was extracted from root and leaf tissues using Plant RNA Kit (Omega, London, UK), and then was subjected to reverse transcription reaction with the RevertAid First Strand cDNA Synthesis Kit (Thermo Scientific, USA) to synthesize the first cDNA strand according to the manufacturer’s protocol. The divergent PCR primers were designed using the “out-facing” strategy to guarantee the amplifications were from circle template. Primers were synthesized by Sangon Biotech Co., Ltd. (Shanghai, China). Sanger sequencing (BGI Co., Ltd., Wuhan, Hubei, China) were performed to validate the presence of the back-spliced junction sites. Quantitative real-time PCR (qRT-PCR) was performed using SYBR Green Master Mix (Vazyme, Nanjing, China) on a CFX 96 Real-Time PCR system (Bio-Rad) to validate the expression levels of circRNAs and correspondence parental genes according to reported methods [33]. The relative quantity was calculated with 2^–ΔΔCt^ method [38]. Each sample contains three replications, and each replication contains two technical replications. Primers for these genes were designed using the Primer Premier 5 software and showed in Additional file 3: Table S3.

### Proline measurements, enzymatic activity and qRT-PCR assay

Proline content was measured spectrophotometrically using the ninhydrin assay [39]. The activities of proline dehydrogenase (PDH) and D1-pyrroline-5-carboxylate synthetase (P5CS) were determined according to Singh, et al. [40]. Protein in the dialyzed extracts was measured colorimetrically at 595 nm based on the formation of protein-dye complex (the binding of Coomassie Brilliant blue G-250 to protein) as described by Bradford [41] using bovine serum albumin (BSA) as the standard. RNA extraction and quantitative qPCR analysis of the expression level of PDH (Csa5G643300) P5CS (Csa3G185140) were performed as described above with gene-specific primers (Additional file 3: Table S3).

## Results

### Plant growth parameters under control and salt stress conditions

**Fig. 1:**
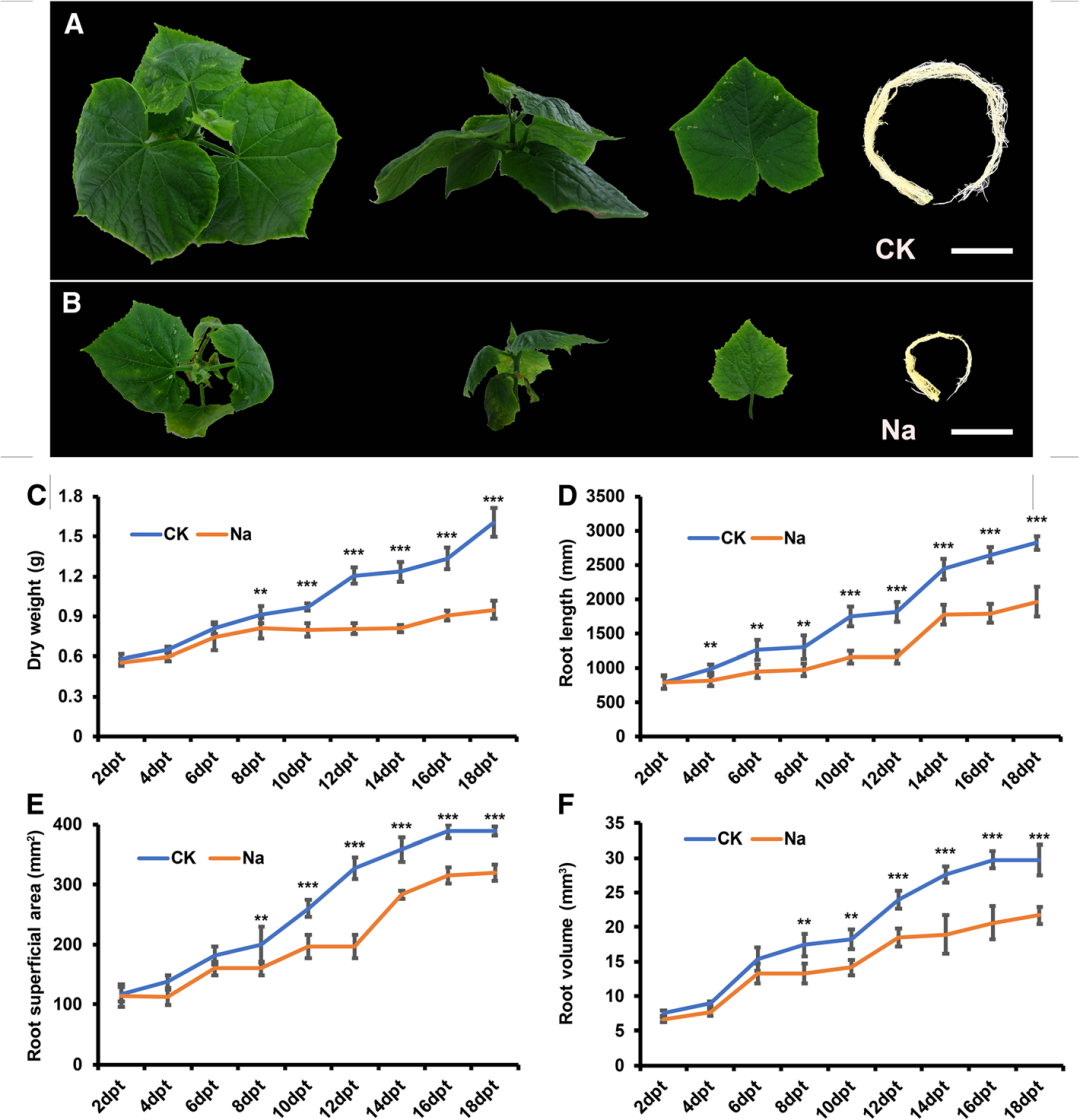
Cucumber dry weight and root traits under control and salt stress conditions. Plant growth under **a** control (CK) and **b** 75 mM NaCl treatment. **c** Plant dry weight and **d** root length, **e** superficial area, and **f** volume. Values are presented as means of six replicates ± SE. CK, control; Na, salt stress. ** *p* < 0.01. dpt, the days post of treatment. Bars = 5 cm

As can been seen in Fig. 1, salt stress significantly decreased plant dry weight, root superficial area, and root volume after 8 days of treatment. Root length was decreased by salt stress after 4 days of treatment compared with control (Fig. 1). Since the growth parameters have shown a downward trend in NaCl-treated seedlings compared with CK after 4 days of treatment, the 3 days’ treatment seedlings were used to identify circRNAs in cucumber because we were particularly interested in the early responses to salinity.

### Identification of circRNAs in cucumber

**Fig. 2:**
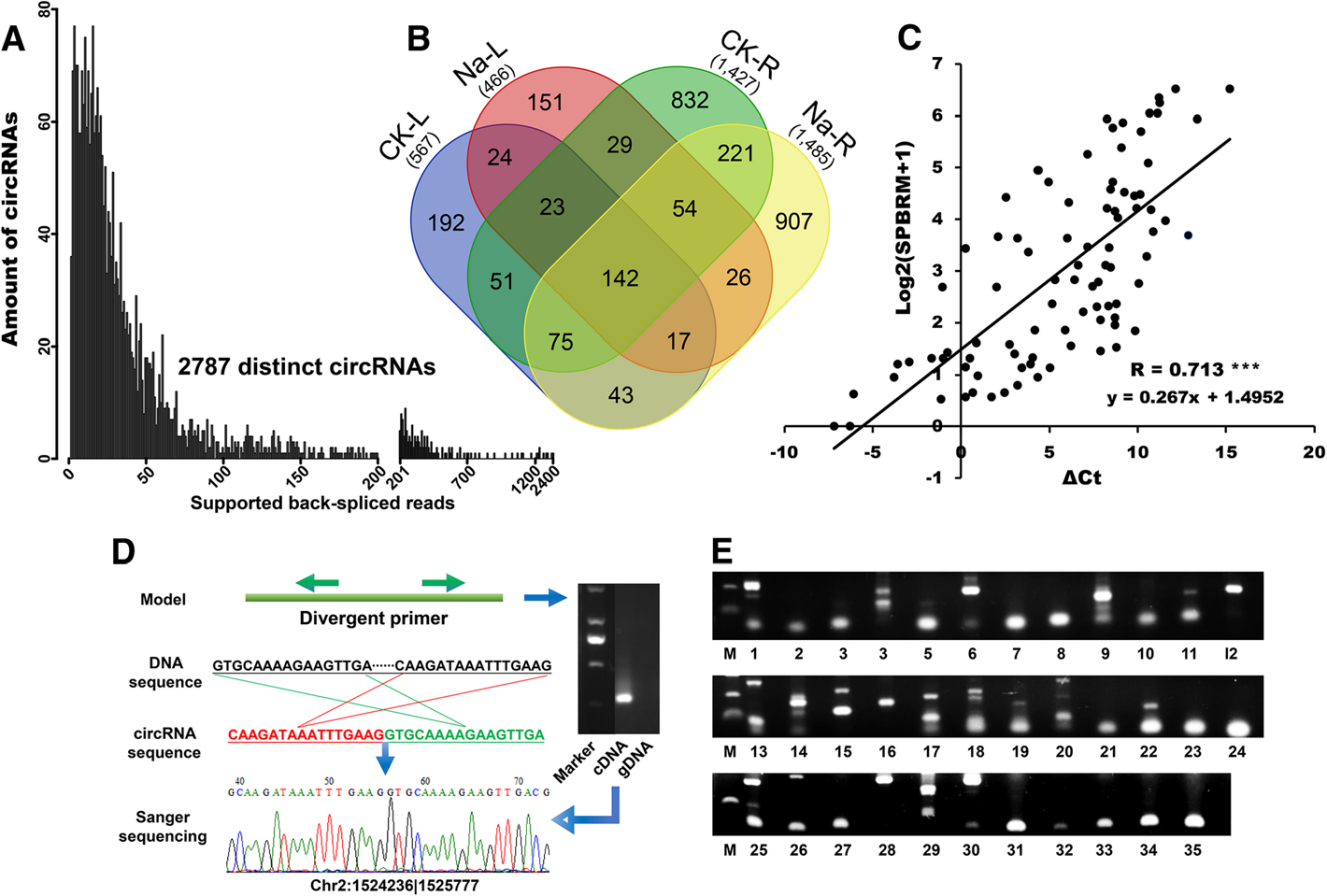
The distribution of circRNAs in different tissues and treatments and circRNAs validation. **a** Detected amount of circRNAs and their cognate supporting back-splicing junction reads. **b** Venn diagram showing the amount and distribution of circular RNAs in each tissues of cucumber under control and salt stress conditions. **c** Correlation of the abundance levels of circRNAs and their corresponding parental genes in cucumber. **d** Validation of cucumber circRNA through PCR and Sanger sequencing using Chr2:1524236|1525777 as an example. Upper left panel (Model), a model used to show the divergent primers for circRNAs backsplicing sites amplification. Upper right panel, an example showing that amplification of divergent primers can be amplified from cDNA but not from genomic DNA. Two middle panels, respectively, black rectangle (DNA sequence) representing downstream and upstream sequences in the genome, and red and green rectangles (circRNA sequence) representing back-splicing circRNA sequence. Lower panel, a Sanger sequencing example of cucumber circRNA Chr2:1524236|1525777. **e** M, marker; 1 to 35, thirty-five circRNA validation using Sanger sequencing. (Detailed information, including the corresponding ID of 1 to 35 circRNAs and their divergent primers, can be found in Additional file 3: Table S3)

To identify cucumber circRNAs, we extracted total RNA from leaf (L) and root (R) tissues under both control and salt stress conditions, constructed ribosomal RNA- and liner RNA-depleted RNA libraries, and deeply sequenced the libraries using Illumina Hiseq X-ten platform. In total, about 450 million raw reads were obtained from eight samples (CK-L1, CK-L2, Na-L1, Na-L2, CK-R1, CK-R2, Na-R1, and Na-R2). After removing low quality reads, clean reads were aligned to reference genome using BWA-MEM, about 334 million reads were uniquely mapped (Table 1). Then CIRI2, the circRNA identification tool, was used to inspect the produced SAM files, which resulted in the detection of 154,210 junction reads. Based on the back-spliced reads, 2787 circRNAs were finally detected, with 827 in leaf and 2420 in root (Fig. 2a, Additional file 4: Table S4). Among these 2787 circRNAs, 367 and 1960 circRNAs were specifically expressed in leaf and root, respectively, indicating that circRNAs are tissues specific (Fig. 2b). Besides, by divergent polymerase chain reaction (PCR), 35 out of 40 (87.5%) selected circRNAs were confirmed to be processed from back-splicing, demonstrating the reliability of our high-throughput sequencing based circRNA identification (Fig. 2d, e. Additional file 3: Table S3). The overall abundances of circRNAs in cucumber were positively correlated with their parental gene abundances both in leaf and root (r = 0.713, *p* < 0.005, Fig. 2c).

**Table 1:** Genome-wide identification of circRNAs in cucumber

Sample	Total read	Total nucleotide	Unique mapped read	Junction read	CircRNA
CK-L1	64,941,534	9,730,914,122	59,683,975 (91.90%)	4115 (0.006%)	337
CK-L2	47,247,952	7,078,390,750	43,533,334 (92.14%)	3223 (0.007%)	344
Na-L1	52,711,124	7,897,509,504	48,504,173 (92.02%)	2814 (0.005%)	277
Na-L2	56,139,180	8,410,167,314	51,545,903 (91.82%)	3610 (0.006%)	288
CK-R1	55,226,326	8,245,076,746	37,769,894 (68.39%)	28,936 (0.052%)	807
CK-R2	48,243,900	7,210,362,462	33,538,249 (69.52%)	29,102 (0.060%)	924
Na-R1	55,918,188	8,351,234,926	23,369,033 (41.79%)	45,248 (0.081%)	819
Na-R2	65,369,878	9,767,970,292	36,585,168 (55.97%)	37,162 (0.057%)	909
All	445,798,082	66,691,626,116	334,529,729	154,210	2787

### Characterization of cucumber circRNAs

**Fig. 3:**
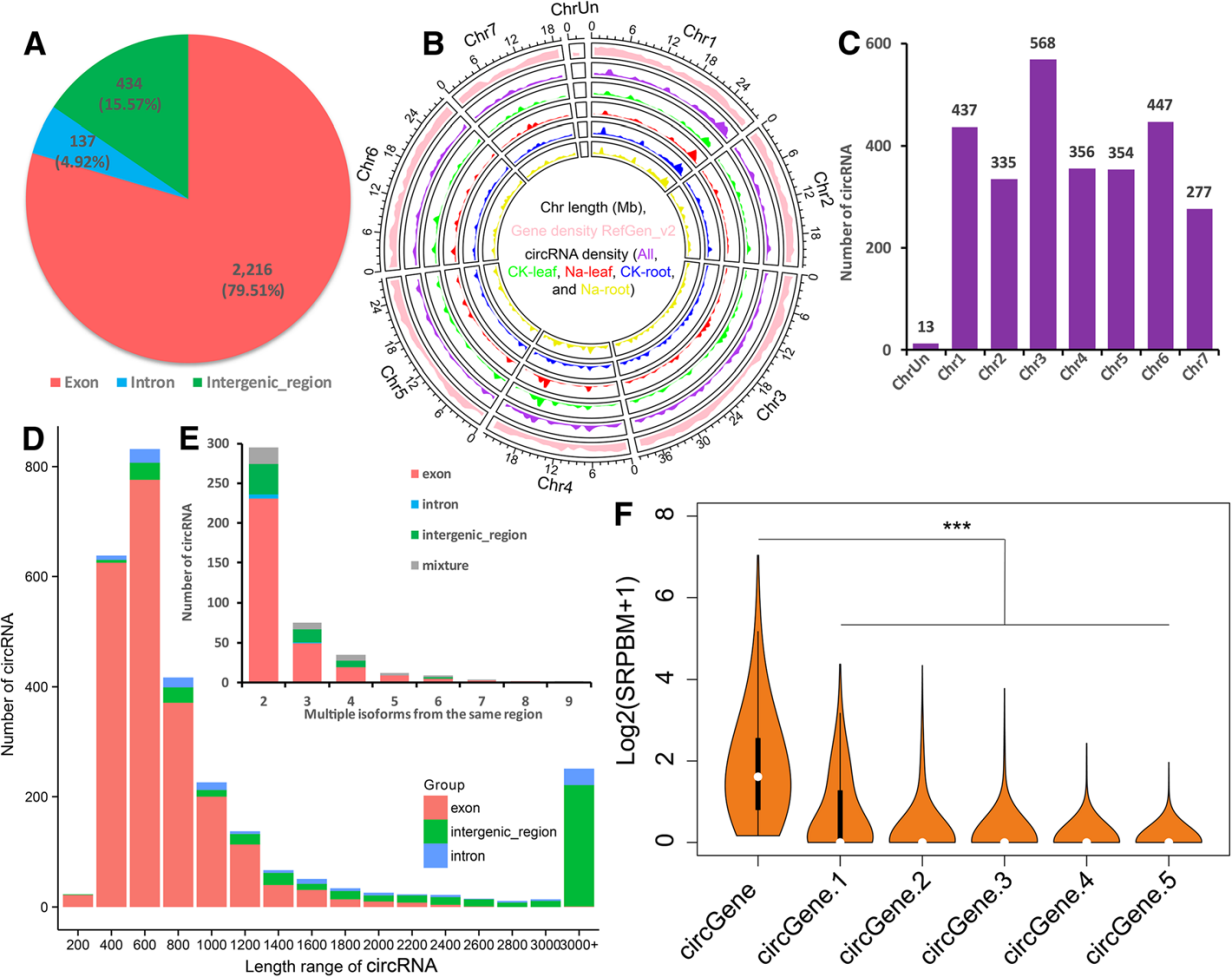
Characterization of cucumber circRNAs. **a** Pie chart representing the amount and percentage of circular RNAs generated from exonic, intronic, and intergenic regions. **b** Circos showing the distribution and density of circular RNAs in seven cucumber chromosomes. **c** Histogram showing the number of circRNAs detected in seven cucumber chromosomes. **d** Histogram showing the length range distribution of cucumber circRNAs. **e** Alternative circularization events statistic. Mixture means alternative splicing events generated from exonic, intronic, and/or intergenic_region. **f** Violin plot describing the comparison of the levels of most abundantly expressed circular RNA isoform (circGene) and other isoforms (circGene.x) from one gene locus (*n* = 1131). The top six high expressed circRNAs were presented (*** means Wilcoxon rank-sum test *p* < 0.005)

Genomic origin analysis showed that the largest majority (2216, 79.51%) of the 2787 identified circRNAs were exonic circRNAs, 434 (15.57%) were intergenic circRNAs, and the rest 137 (4.92%) were intronic circRNAs (Fig. 3a). Genomic density analysis suggested that these cucumber circRNAs were distributed across the whole genome. Nevertheless, similar to coding genes, circRNAs were more commonly found distribution at both ends of chromosomes (Fig. 3b). As to chromosome distribution, the most circRNAs were found in chromosome 3, followed by chromosome 6 and 1 (Fig. 3c). Considering the length, most circRNAs (*n* = 2391; only known splice lengths without introns) are shorter than 1600 nucleotide (nt), the median length is ~ 570 nt, and large majority of long circRNAs are intergenic type (Fig. 3d. Additional file 4: Table S4). Alternative circularization analysis showed that 1131 alternative back-splicing circularization events were detected from 434 unique chromosome loci, of which, 319 from exon region, 66 from intergenic region, 6 from intron region, and 43 from mixture regions (including exon, intron and/or intergenic types). Statistically speaking, 295 of 434 loci produced two distinct circRNA isoforms, 75 produced three isoforms, 35 produced four isoforms, 12 produced five isoforms, and 2 regions produced nine different circRNAs isoforms (Fig. 3e, Additional file 4: Table S4). Then, we investigated the expression abundance of the circRNA isoforms within one gene locus and found that about 74.71% parental genes (845 in 1131, circRNAs without isoforms were excluded in the analysis) produced a significantly higher expressed circRNA (at least twofold higher than other isoforms) (Fig. 3f). These results indicate that there is often a predominantly expressed circRNA isoform from one gene locus.

To explore conserved features of circRNAs in different plant species, the circRNAs originated from the cognate orthologous genes of *Arabidopsis thaliana*, *Oryza sativa*, *Glycine max*, *Zea mays*, *Solanum lycopersicum*, *Solanum tuberosum*, *Triticum aestivum* and cucumber were compared. circRNAs belonging to these species were downloaded from the PlantcircBase [42]. The orthologous gene sets between cucumber and these species were generated by BioMart from EnsemblPlants database. Among the 8362, 11,147, 3876, 2209, 984, 260, 31, and 1642 parent genes that produced exonic circRNAs in Arabidopsis, rice, soybean, maize, tomato, potato, barley, and cucumber, 1493 gene pairs between cucumber (1210 unigenes) and Arabidopsis (1462 unigenes), 1459 gene pairs between cucumber (1075 unigenes) and rice (1422 unigenes), 897 gene pairs between cucumber (656 unigenes) and soybean (877 unigenes), 526 gene pairs between cucumber (451 unigenes) and maize (502 unigenes), 241 gene pairs between cucumber (234 unigenes) and tomato (234 unigenes), 24 gene pairs between cucumber (24 unigenes) and potato (24 unigenes), and 6 gene pairs between cucumber (6 unigenes) and barley (5 unigenes) were orthologs (Additional file 5: Table S5), accounting for 17.48, 12.76, 22.63, 22.73, 22.78, 9.23, 16.13, and 95.83% of Arabidopsis, rice, soybean, maize, tomato, potato, barley, and cucumber genes producing exonic circRNAs, respectively. Furthermore, orthologous genes of 1175 cucumber parental genes (1175/1642, 72%) were found in more than 2 species, while 286 of that (286/1642, 17%) were found in 1 species and no orthologous genes of 181 cucumber parental genes were detected from other species (Additional file 6: Figure S1). Meanwhile, according to reciprocal BLAST search analysis, 257 cucumber circRNAs had significant alignments with 1540 circRNAs obtained from 12 species in PlantcircBase (e value <1e-5). The nucleotide identity levels between 257 cucumber circRNAs and significant alignments were > 70%. These results further suggested the conservation feature of cucumber circRNAs.

### Orientation-opposite complementary sequences are not preferentially flanked by circularized exons

**Fig. 4:**
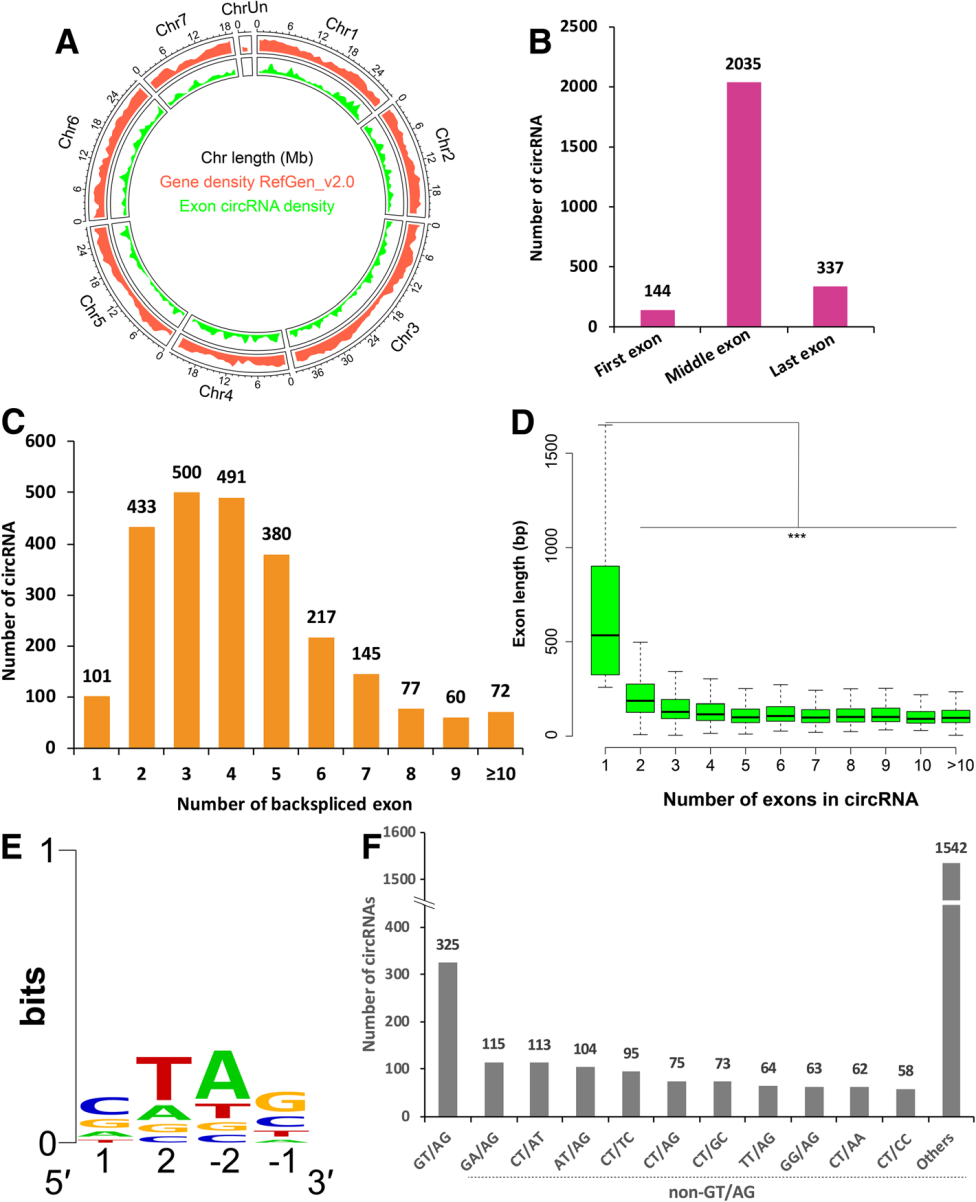
Feature analyses of genomic exon circRNAs. **a** Distribution of backsplicing exons across the reference genomic. **b** Most backsplicing exons prefer to locate in the middle of parental genes, and very few exons are the first or the last parental gene exons. **c** Distribution of backsplicing exons number. The vast majority (about 96%) of circular RNAs contain multiple backsplicing exons. **d** Length distribution of backsplicing exons in circRNAs. Box plots reveal that the distribution of exon length (y axis) is correlated with the numbers of backsplicing exons (x axis). Backsplicing exons in these circRNAs with less exons are usually longer. ***p < 0.005, Wilcoxon rank-sum test. **e** A consensus logo derived from the data is also shown (generated using WebLogo). **f** Splicing signals of circRNAs identified in our circRNA-seq dataset from cucumber roots and leaves. In the non-GT/AG group, each splicing site of the top 10 splicing signals were individually showed, and “Others” represents the total number of remaining non-GT/AG splicing sites of circular RNAs

To further reveal the possible biosynthesis mechanisms of exon circRNAs, we then investigated the genomic features of more confidence exon circRNAs with high expression levels (circRNAs supported by at least five junction reads) (Fig. 4a). As can been seen in Fig. 4b, among the highly expressed exonic circular RNAs identified in this study, the vast majority were processed from those exons distributed in the middle of parent genes (2035), and only a few circRNAs contain the first (144) or the last exons (337) (Fig. 4b), implying that the formation of circular RNAs is commonly coupled with RNA splicing process. Moreover, most circular RNAs have multiple exons (generally 2–5 exons), while 101 out of 2035 highly expressed circular RNAs contain only one annotated exon (Fig. 4c). Interestingly, these exons from circular RNAs comprised by only one circularized exon were much longer than exons from circular RNAs containing multiple circularized exons, revealing that a certain length of exon(s) was probably preferred by exon circRNAs to maximize the circularization (Fig. 4d). Several studies proposed that, compared to animals, plant (e.g. *Arabidopsis thaliana*, rice and soybean) exonic circRNAs are less likely to be generated from exon(s) flanked by introns containing repetitive and/or reverse complementary sequences [10, 15, 43]. To explore whether or not cucumber is the same, we analyzed the flanking sequences of exon circRNAs and found that only 18 circRNAs contain at least 18-bp flanking complementary sequences at both end of 1000-bp flanking sequences (Additional file 7: Table S6). Meanwhile, miniature invertedrepeat transposable elements (MITEs) searching found that only 6 circRNAs have two flanking sequences that overlapped with MITEs. These results confirmed that the circularization of exon circRNAs in cucumber maybe not mediated by flanking orientation-opposite complementary sequences. In both plants and animals, U2-dependent spliceosome is involved in the splicing of vast majority of introns with 5’-GT and 3’-AG terminal dinucleotides [21]. To confirm whether the 5’-GT and 3’-AG are the circRNA splicing signal in cucumber, the splice sites of 2787 cucumber circRNAs were further analyzed and we found that the splicing sites were dominated by C and T nucleotide in 5’ termini and A and G nucleotide in 3’ termini (Fig. 4e). And only a minority portion (325 or 11.7%) were flanked by the common GT/AG (including CT/AC equivalent) splicing signals (Fig. 4f). Moreover, a diverse set of non-GT/AG splicing signals were also found and the top five non-GT/AG terminal dinucleotides pairs were GA/AG (115), CT/AT (113), AT/AG (104), CT/TC (95), and CT/AG (75) (Fig. 4f). Accordingly, our analysis demonstrates that, except the canonical GT/AG, cucumber circRNAs also use diverse non-GT/AG splicing signals for circularization.

### Differentially expressed circRNAs induced by salt stress

**Fig. 5:**
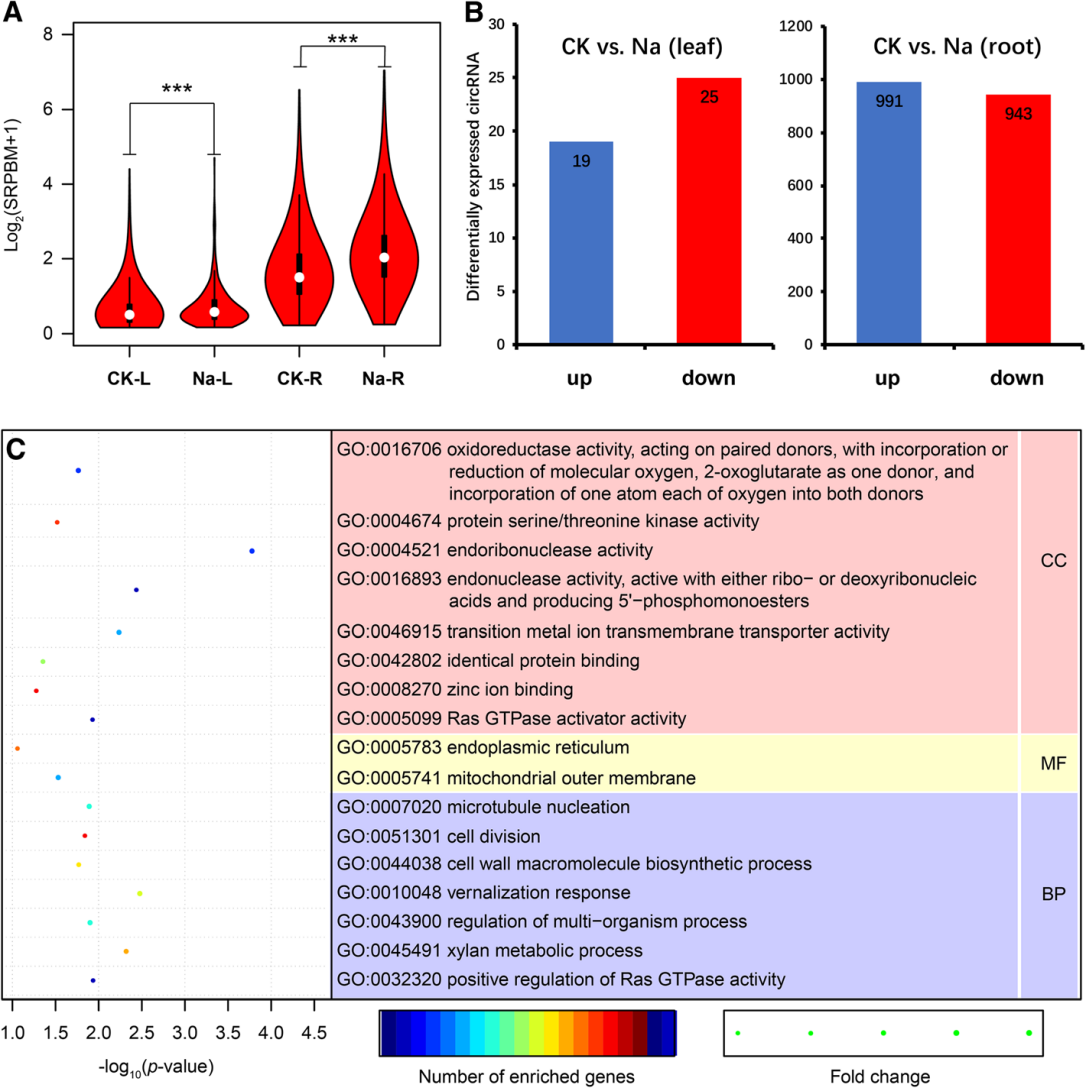
Differentially expressed circRNAs in response to salt stress. **a** Violin plot of relative abundance of circRNAs in salt stress tissues compared with the paired normal tissues. Data are expressed as the log_2_(SRPBM+ 1). The white dot represents the median. ***p < 0.005, Wilcoxon rank-sum test. **b** Number of differentially expressed circRNAs. **c** GO enrichment analysis of differentially expressed circRNAs in root. GO, Gene Ontology; CC, cellular component; MF, molecular function; BP, biological process

To overview the effect of salt stress on cucumber circRNAs, we pair-wised compared the whole expression levels of all detected circular RNAs. Wilcoxon rank-sum test indicated that circRNAs are significantly up-regulated by salt stress in both tissues (leaf, *P* < 6.07e-06; root, *P* < 2.2e-16. Figure 5a). In order to reveal cucumber circRNAs that have biological functions in responding to salt stress, we compared the expression profiles of differentially expressed circRNAs between control and salt stress treatments. Among the 2787 circRNAs, 1966 circRNAs were significant differential expressed under salt stress, including 1934 in root (991 up- and 943 down-regulated, Additional file 8: Table S7) and 44 in leaf (19 up- and 25 down-regulated, Additional file 9: Table S8) (Fig. 5b).

### Functional categorization of stress-regulated parent genes

#### Functional classification by GO annotation

Since circRNA function could be related to the known function of the host linear transcripts [1, 3], we predicted and annotated the parent genes of regulated circRNAs. GO enrichment analysis showed that, in root, GO terms related to: (1) Biological Process (BP), including positive regulation of Ras GTPase activity (GO:0032320), xylan metabolic process (GO:0045491), regulation of multi-organism process (GO:0043900), vernalization response (GO:0010048), cell wall macromolecule biosynthetic process (GO:0044038), cell division (GO:0051301), and microtubule nucleation (GO:0007020); (2) Cellular Component (CC), including mitochondrial outer membrane (GO:0005741), and endoplasmic reticulum (GO:0005783); (3) Molecular Function (MF), including Ras GTPase activator activity (GO:0005099), zinc ion binding (GO:0008270), identical protein binding (GO:0042802), transition metal ion transmembrane transporter activity (GO:0046915), endonuclease activity, active with either ribo- or deoxyribonucleic acids and producing 5′-phosphomonoesters (GO:0016893), endoribonuclease activity (GO:0004521), protein serine/threonine kinase activity (GO:0004674), and oxidoreductase activity, acting on paired donors, with incorporation or reduction of molecular oxygen, 2-oxoglutarate as one donor, and incorporation of one atom each of oxygen into both donors (GO:0016706), were specifically enriched (Fig. 5c, Additional file 8: Table S7). Similar results were also reported in previous studies, which suggested that plant salt tolerance is a complex regulating network involving in signal transduction, and substance and energy metabolism [44, 45].

#### Genes related to abiotic stress responses and plant development

In leaf, the expression of seven annotated circRNAs, including Chr1:3244941|3,245,447, Chr1:20336460|20,339,620 Chr1:21205717|21,206,424, Chr1:25970551|25,973,020, Chr3:10198472|10,200,676, Chr4:23091126|23,091,996, and Chr6:9785683|9,788,312, were significantly down-regulated (Table 2). Simultaneously, the parent genes of these seven circRNAs have been proved to be vital for regulating abiotic stress responses and plant development. For example, protein phosphatase 2C (PP2C), members of major phosphatase class, have been reported to regulate abiotic stress triggered signaling pathways. In Arabidopsis, overexpression of *OsPP108*, a group A type PP2C from rice, increased the plant’s insensitive to ABA and tolerance to high salt and mannitol stresses during seed germination, root growth, and overall seedling growth [40]. Plant growth is mainly the results of cell division and elongation, while salt stress inhibits cell division and cell wall metabolism [44, 46]. A decrease in cell wall extensibility has been suggested to be responsible for leaf growth reduction under salt stress [47, 48]. In this research, parent gene of circRNA Chr2:226834|229,565, annotated as type II inositol 1,4,5-trisphosphate 5-phosphatase FRA3-like, has been reported to plays an essential role in the secondary wall synthesis in fiber cells and xylem vessels [49]. Another gene, annotated as molybdenum cofactor sulfurase (parent gene of circRNA Chr6:9785683|9,788,312), were found to be an important factor in regulating the ABA levels in plant tissues in response to drought, salt, or ABA treatment [50–52].

**Table 2:** List of selected differentially expressed circRNAs under salt stress in the leaf

circRNA	Parent gene	Log2FC	FDR	Annotation
Chr1:3244941|3245447	Csa1G031190	−3.27	0.046	Kinase superfamily protein isoform 7
Chr1:20336460|20339620	Csa1G560670	−3.27	0.046	Protein transport protein Sec24
Chr1:21205717|21206424	Csa1G571280	−3.49	0.037	Glutathione synthetase, chloroplastic-like
Chr1:25970551|25973020	Csa1G652290	−3.18	0.050	Type II inositol 1,4,5-trisphosphate 5-phosphatase FRA3-like
Chr3:10198472|10200676	Csa3G150160	−3.42	0.040	Glutamine synthetase leaf isozyme, chloroplastic-like
Chr4:23091126|23091996	Csa4G664270	−3.74	0.029	Protein phosphatase 2C 27-like
Chr6:9785683|9788312	Csa6G139140	−3.74	0.029	Molybdenum cofactor sulfurase-like

Similar to leaf, in the roots, several parent genes of differentially expressed circRNAs that belong to protein serine/threonine kinase activity (GO:0004674) including SNF1-related kinases (SnRKs) and PP2C, and cell process like 7 metabolic process (GO:0045491) and cell division (GO:0051301) were obtained (Table 3). As signal transductors, serine/threonine kinases involve in multiple stress tolerance in plants, including salt, drought, and freezing stresses [53, 54]. Moreover, a number of genes related to cell biogenesis including xyloglucan glycosyltransferase and sucrose synthase were identified (Table 3, Additional file 8: Table S7). Thus, a hypothesis was raised that these circRNAs were valuable parts in the signaling transduction pathways and cell wall biosynthesis and modification process through regulating the expression level of their parent genes.

**Table 3:** List of selected differentially expressed circRNAs under salt stress in the root

circRNA	log2FC	FDR	parent gene	annotation
protein serine/threonine kinase activity (GO:0004674)				
Chr1:21522408|21523334	3.64	0.007	Csa1G574260	serine/threonine-protein kinase CTR1-like
Chr3:29130864|29133923	5.256	0.01	Csa3G749850	serine/threonine-protein kinase CTR1-like
Chr3:20613886|20615220	−5.32	0.003	Csa3G444590	proline-rich receptor-like protein kinase PERK8-like
Chr6:5205917|5207198	4.82	0.010	Csa6G077450	SNF1-related protein kinase catalytic subunit alpha KIN10-like
Chr6:10126493|10128076	−5.71	0.002	Csa6G146930	receptor-like serine/threonine-protein kinase NCRK-like
xylan metabolic process (GO:0045491)				
Chr2:2178032|2180266	6.64	0.002	Csa2G012150	5’-3’ exoribonuclease 4-like
Chr3:6464544|6464882	3.94	0.021	Csa3G118000	E3 ubiquitin-protein ligase BRE1-like
Chr5:22801672|22802005	−4.43	0.008	Csa5G606350	galactoside 2-alpha-L-fucosyltransferase-like
cell division (GO:0051301)				
Chr7:16395598|16399679	−6.01	0.001	Csa7G427110	actin-related protein 7-like
Chr4:20046421|20047663	−4.99	0.004	Csa4G622880	α, α-trehalose-phosphate synthase [UDP-forming] 1-like
Chr4:18459900|18468401	6.34	0.003	Csa4G561190	cyclin-dependent kinase E-1-like
Chr4:15928017|15929087	5.59	0.005	Csa4G425740	G2/mitotic-specific cyclin S13–6-like
Chr4:21955412|21956601	−6.01	0.001	Csa4G646110	ATP-dependent zinc metalloprotease FTSH 4
Ras GTPase activator activity (GO:0005099)				
Chr2:21079250|21079598	−4.68	0.006	Csa2G406820	TBC1 domain family member 2A-like
Chr2:8079362|8082138	−6.23	0.001	Csa2G120950	TBC1 domain family member 13-like
Chr3:4284168|4285558	5.26	0.007	Csa3G078290	GTPase-activating protein gyp7-like
Chr4:1337637|1340074	4.29	0.016	Csa4G008220	ADP-ribosylation factor GTPase-activating protein AGD3-like
Chr6:26240476|26241992	5.14	0.008	Csa6G510340	Ras-related protein RABB1c-like
Ion homeostasis				
Chr2:16186442|16191680	6.44	0.003	Csa2G351730	K^+^ efflux antiporter 6-like
Chr3:25629954|25631297	5.14	0.008	Csa3G651710	vacuolar cation/proton exchanger 2-like
Chr3:7346435|7347924	5.20	0.008	Csa3G122590	K^+^ efflux antiporter 2, chloroplastic-like
Chr5:2818033|2823737	−7.03	0.000	Csa5G098980	sodium/hydrogen exchanger 7-like

#### Genes related to the ion transport

The ability of a plant to maintain a high cytosolic K^+^/Na^+^ ratio is critical to plant salt tolerance. Na^+^/H^+^ (NHX) antiporters and K^+^ transporter have important role in cellular Na^+^, K^+^ homeostasis [19, 55]. In this study, the expression level of four circRNAs, Chr3:25629954|25,631,297, Chr2:16186442|16,191,680, Chr3:7346435|7,347,924 and Chr5:2818033|2,823,737, were significantly affected by salinity (Table 3). Their parent genes were predicted to encode a vacuolar cation/H^+^ exchanger 2-like (CHX2-like), K^+^ efflux antiporter 6-like (KEA6-like), K^+^ efflux antiporter 2-like protein (KEA2-like), and sodium/hydrogen exchanger 7-like protein, (NHX7-like), respectively. NHX7, also known as *SOS1*, is a member of vacuolar NHX, and has been proved to improve salt tolerance through reducing Na^+^ accumulation in the shoot of *Arabidopsis thaliana* [56]. The functions of NHX and KEA transporters are involved in K^+^ and H^+^ movements across endomembranes, and it is becoming more and more accepted that the expression level of these transporters are regulated by NaCl and osmotic stress [57, 58]. However, the detailed function of these antiporters in K^+^ homeostasis is poorly understood [57, 59]. These results in our study might have implications for the role of circRNAs related to Na^+^/H^+^ (NHX) antiporters and K^+^ transporters in cucumber and could be helpful to further discovered the mechanisms of the maintenance of optimal K^+^/Na^+^ ratios under saline conditions.

### circRNA-miRNA-mRNA regulating network

#### Functional classification by eggNOG annotations

**Fig. 6:**
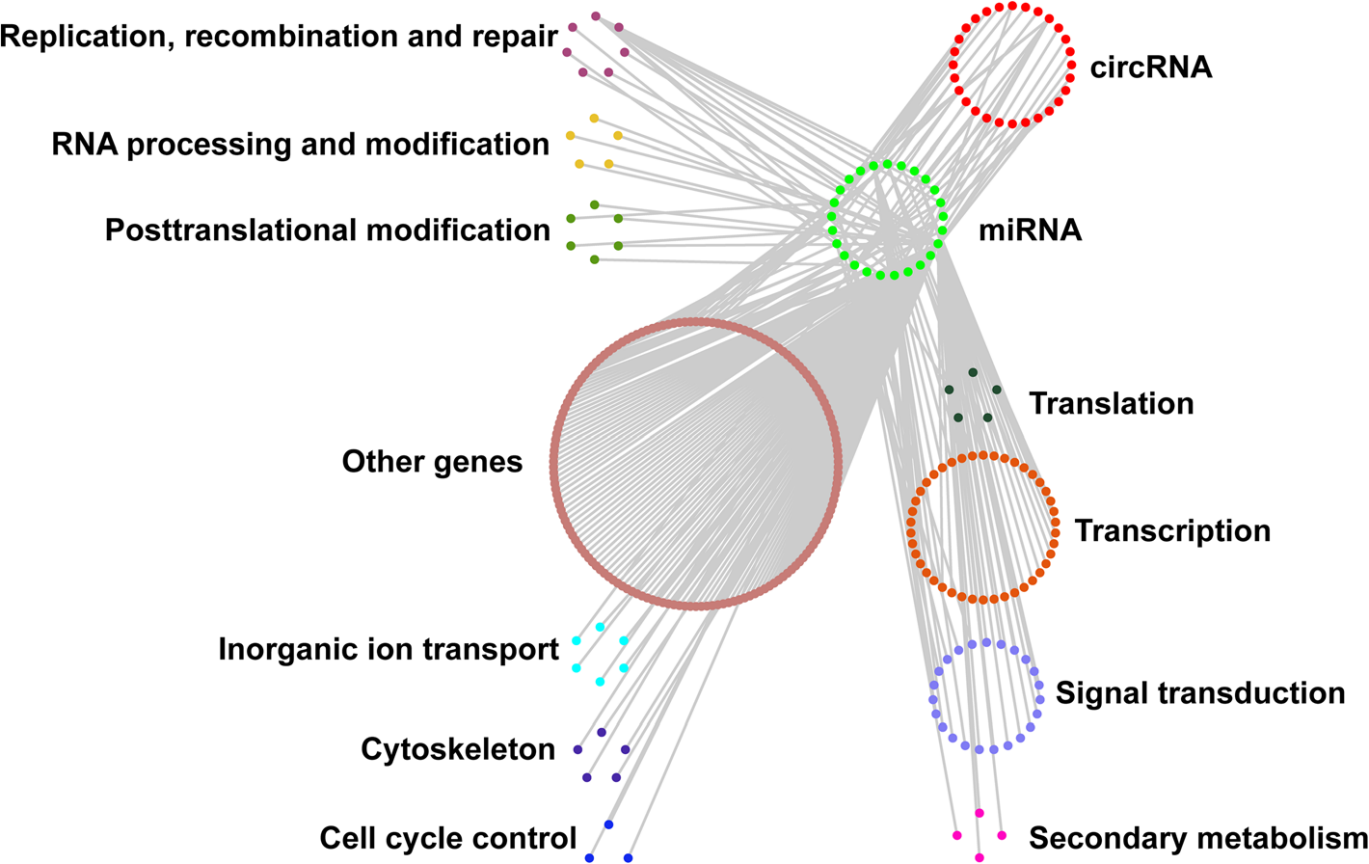
Possible regulatory networks of cucumber responding to salt stress. The networks include differentially expressed circRNAs and their target genes. Red nodes represent the circRNAs, lima nodes represent the miRNAs, and other colored nodes represent mRNAs, respectively. Detailed information, including binding sites, relationship among circRNAs, miRNAs, and mRNAs, and annotation of mRNAs were listed in Additional file 10: Table S9, Additional file 12: Table S12, Additional file 13: Table S10

Besides *cis*-regulating parent genes, circRNAs showed an ability to act as competing endogenous RNAs [60]. To explore the potential functional importance of circRNAs by binding to miRNA and further affect the post-transcriptional regulation of genes, we construct the circRNA-miRNA-mRNA network in cucumber. Among the 1966 differentially expressed circRNAs, 30 circRNAs were found to contain miRNA identical regions, implying that these circRNAs may have miRNA binding sites and could function as miRNA sponges, which need to be validated experimentally. Target prediction showed 45 miRNAs to be the potentially binding targets of these 30 circRNAs (Additional file 10: Table S9). Moreover, 269 mRNAs were predicted to be the potential targets of 26 miRNAs (Additional file 11: Table S11, Additional file 12: Table S12). The functions of 269 cucumber genes which transcript the potential target mRNAs were further analyzed by eggNOG class annotations analysis. Just like the GO terms analysis to circRNA parent genes, iron transporting and cell cycle controlling were obtained (Fig. 6).

#### Genes related to transcription factors

Besides, a lot of genes involved in transcription regulations and signal transduction were obtained, which has been reported to play important roles in response to environmental stresses [61]. To address complicated environmental stresses, many transcription factor (TFs) genes are activated in plant cells through signal perception and subsequent signal transduction [62]. Accordingly, in this study, many of the predicted miRNA targets are various TFs, including ethylene-responsive TFs, bZIP, MYB, SBP and ZF-HD (Additional file 10: Table S9). In plants, Ethylene is an important inducer of defence-related genes in both biotic and abiotic stresses [63, 64]. In this study, four RAP2-like ethylene-responsive transcription factors (Csa3G736760, Csa4G292470, Csa5G175970, and Csa6G296960) were predicted to be the targets of scaffold01066:88458_88477. It is reported that RAP2-like ethylene-responsive transcription factor participates in ABA, salt and osmotic stress responses in Arabidopsis [65]. Moreover, ERF054-like ethylene-responsive transcription factor (Csa6G128610) and CRF4-like ethylene-responsive transcription factor (Csa5G139630) were predicted to be the targets of csa-miRn6-3p (Table 4. Additional file 10: Table S9). ERF054-like ethylene-responsive transcription factor is found to involved in salt stress response in radish (*Raphanus sativus* L.) and cold stress response in *Rosa multiflora* [66, 67]. Further studies are required to explore their exact roles in cucumber salt stress adaptation.

**Table 4:** List of selected miRNAs sponged by circRNAs and their target mRNAs in the root

circRNA	log2FC	FDR	sponged miRNA	target mRNA	annotation
Chr1:26661035|26687720	−4.43	0.008	scaffold01066:88458_88477	Csa3G736760	ethylene-responsive transcription factor RAP2-like
Chr1:26677510|26687733	−5.25	0.003		Csa4G292470	ethylene-responsive transcription factor RAP2-like
				Csa5G175970	ethylene-responsive transcription factor RAP2-like
				Csa6G296960	ethylene-responsive transcription factor RAP2-like
Chr3:14927566|14937448	−3.52	0.027	csa-miRn6-3p	Csa6G128610	ethylene-responsive transcription factor ERF054-like
Chr7:14083607|14090637	6.46	0.002		Csa5G139630	ethylene-responsive transcription factor CRF4-like
Chr6:19177739|19183752	−4.43	0.008	miR159b	Csa4G022940	transcription factor MYB29-like
Chr1:1271310|1273631	3.80	0.023	csa-miR-171 l	Csa1G051590	squamosa promoter-binding-like protein 13A-like
				Csa6G094760	squamosa promoter-binding-like protein 16-like
Chr3:14927566|14937448	−3.52	0.027	csa-miRn6-3p	Csa4G377720	protein ABSCISIC ACID-INSENSITIVE 5-like
Chr7:14083607|14090637	6.46	0.002		Csa4G377720	protein ABSCISIC ACID-INSENSITIVE 5-like

MYB transcription factors play impotent roles in diverse processes including developmental control, cell cycle control, secondary metabolism and responses to biotic and abiotic stresses in plant [68]. In this study, several mRNA annotated as MYB-like were identified (Table 4. Additional file 10: Table S9). To be specific, targets of miR159b is functional annotated as MYB29-like transcription factor (Csa4G022940). In Arabidopsis, MYB29 has been revealed to function in the complex interplay of ethylene, jasmonic acid, and salicylic acid, and ROS signaling by regulating the expression of various ethylene responsive factors and WRKY transcription factors [69]. In summary, combining with these former studies, our results reveal that those miRNAs and their corresponding circRNAs playing roles in salt stress responding by remolding the transcript of TFs (Fig. 6).

This circRNA-miRNA-mRNA network has been constructed in many studies related to plant circRNAs. The circRNA-miRNA interaction network could provide valuable candidates for experimentally texting the significant regulatory function by non-coding RNA regulatory network involving circRNAs in plants. However, this was associated with two problems. First, since only a few experimentally proven miRNA targets could be deciphered, it is hard to predict the functions of circRNAs more accurately. Second, unlike in animal, in plant, there is still no experimental evidence proves circRNAs can act as miRNA sponge and Chu, et al. [70] suggested that miRNA sponges might not be the major functional mechanism of plant circRNAs. Thus, we further mined conserved miRNAs, which have been well known to extensively regulate analogous targets and play critical roles in plant development and abiotic stresses [71]. Some well-known miRNA families like miR156, miR159, miR172, miR398 miR399 and miR396 were predicted to be targeted by certain cucumber circRNAs. And most of these miRNAs were proved to be play roles in diverse interconnected biological programs and plays critical roles in responses to biotic and abiotic stresses. (Additional file 13: Table S10). In further studies, these conserved miRNAs can be important candidates to understand the circRNA-miRNA interactions as well as specific functions of these circRNAs in cucumber plant.

### circRNAs mediate proline (pro) metabolisms by regulating corresponding biosynthesis and degradation genes

In this study, the cluster for ‘amino acid transport and metabolism’ was one of the largest groups among the annotated COG categories. In this category, circRNA Chr3:13041746|13,044,121 (corresponding parent gene Csa6G008780) and Chr6:915006|915,916 (corresponding parent gene Csa3G185140) are both significantly up-regulated under salt stress. Csa6G008780 encodes a rate-limiting enzyme involved in the biosynthesis of proline in higher plants, D1-pyrroline-5-carboxylate synthetase (P5CS). Csa3G185140 (homolog to Arabidopsis Drought and Freezing Responsive gene 1) has been reported to positively regulate proline accumulation through interacts with proline degradation enzymes PDH and P5CDH and compromises their activities in Arabidopsis [72]. Further, we test whether circRNAs participate in proline synthesis and degradation regulation in physiological level. To this end, the expression level of Csa6G008780, Csa3G185140, and the activity of P5CS, PDH were determined. As a result, in the synthesis pathway, circRNA Chr6:915006|915,916 is significantly down-regulated whereas the expression level of P5CS (Csa6G008780) is significantly increased under salt stress. Accordingly, proline content and P5CS activity are significantly increased. Proline content is determined by proline synthesis and degradation. In the degradation pathway, the expression level of Chr6:915006|915,916 and Csa3G185140 is significantly up-regulated while *PDH* (Csa5G643300) expression level and PDH activity were significantly downregulated by salt stress, which is in consistent with the incensement of proline content under salt stress. These results suggested that Chr6:915006|915,916 may be response to salt stress and positively regulate Csa3G185140, which may also be responsible for the decrease of *PDH* (Csa5G643300) expression level and eventually PDH activity. Our results suggested that Chr3:13041746|13,044,121 and Chr6:915006|915,916 may act as a negative and positive regulator of their parent genes, respectively, which need to be further confirmed in the molecular level.

## Discussion

Circular RNAs (circRNAs) are a class of special RNAs generated by a non-linear backsplicing event between a downstream splice donor and an upstream splice acceptor. Recently, with the development of circRNA-seq, a huge number of circRNAs have been identified in both animals and plants. The discovering of these widely expressed and highly conserved circRNAs has increased the potential impact of non-coding RNAs on cellular function [73]. Compared with animals, the biogenesis, regulation, and function of circRNAs are largely unknown in plants [12]. Ye, et al. [10] characterized circRNAs in *Oryza sativa* and *Arabidopsis thaliana* and reported that the biogenesis mechanisms of circRNAs from plants could be different from those in animals. Further studies showed that plant circRNAs are conserved, expressed at low concentrations, and in a tissue-specific manner. Moreover, recent studies in rice, wheat, kiwifruit, and potato demonstrated that the circRNAs play an important role in regulating the response to environmental stress such as phosphate imbalance, dehydration stress, and pathogen invasion [10, 12–14]. Cucumber is an important horticultural crop and is fairly sensitive to salinity. To our knowledge, there is still no evidence showing the role of circRNAs in salt-stressed cucumber.

In the present study, we reported the first genome-wide identification and characterization of circRNAs in cucumber and the potential regulation roles of circRNAs under salt stress condition. circRNAs can derive from both root and leaf. In total, 2787 circRNAs were detected, including 827 from leaf and 2420 from root (Table 1. Additional file 4: Table S4). In consistent with previous studies, we found that circRNAs are tissues specific, with 367 and 1960 specifically expressed in leaf, and root, respectively [15, 74]. The circRNAs identified in cucumber was less than that in *Oryza sativa* (26,160), *Arabidopsis thaliana* (31,079), soybean (5372), and kiwifruit (3582), but more than that in wheat (88), maize (496), barely (47), tea (342), and tomato (854), which may due to the difference in species, methods (e.g. experimental strategy or bioinformatic method), tissues (e.g. leaf, root, fruit, stem, shoot), and circRNA prediction tools (e.g. CIRI2, fnd_circ, CIRCexplorer), that are used in different researches [75]. Besides, by divergent PCR, 35 of these circRNAs were validated to be processed from back-splicing (Fig. 2d, e and Additional file 2: Table S2), confirming the reliability of our circRNA-seq results (Additional file 4: Table S4). This large amount of circRNAs detected in this study indicated that circRNAs may contain one of the largest RNA families in cucumber transcription. It also increased the potential impact of non-coding RNAs on cell function and the complexity of regulatory processes [73]. Examining the roles of individual circRNAs will be the subject of future investigations.

CircRNAs can be produced from exons (exonic circRNA), introns (intronic circRNA), and intergenic regions of the genome [1]. In rice, Arabidopsis, tea and tomato, most identified circRNAs were exonic circular RNAs [10, 16, 43]. Similarly, in this study, the majority of the identified circRNAs (2216, 79.51%) were exonic circRNAs (Fig. 3a). However, in wheat and kiwifruit, 51 and 60.2% of the circRNAs were intergenic circRNAs, respectively. In soybean, most of the total circRNAs (in leaf, root, and stem) were intronic circRNAs, while there are more intronic in the root and more exonic in the stem [15]. Low percentage of exonic circRNAs in wheat might due to the huge genome size with comparable fewer available genes and higher percentage of introns circRNAs in soybean might be attributed to the large and duplicated genome and multiple copies of genes [14, 15]. Integrating, these results indicated that the molecular basis of circular RNA biogenesis in plants is quite complicated, which is species- and tissue-specific [13–15]. Since biogenesis of circRNAs have close relationship with their structure, we then explored the characteristics of cucumber circular RNAs.

The different isoforms of circRNAs are results of alternative back-spliced circularization. In human, studies have shown that exon circularization depends on flanking intronic complementary sequences. Further evidence suggested that alternative formation of inverted repeated *Alu* pairs and the competition between them can lead to alternative circularization. Our results revealed that most alternative circularization events were located in the exon regions, but, interestingly, most alternative circularization with more than 3 isoforms were located in the intergenic regions (Fig. 3e). Besides, chromosome 3, the longest one in cucumber genome, produced more circRNAs than others. Further analysis revealed that circRNAs numbers are significantly correlated with chromosome length (Additional file 14: Figure S2, R^2^ = 0.9298 ***). Interestingly, these exons detected in circRNAs with only one circularized exon are much longer than these exons in circRNAs with multiple circularized exons (Fig. 4d), revealing that circRNAs processing may prefer a certain length to maximize exon(s) circularization. Similarly, most circular RNAs in H9 human embryonic stem cells contain two to three exons and circular RNAs with only one circularized exon were much longer than those from circular RNAs with multiple circularized exons [76]. But whether it is a common character need to be further confirmed in both animal and plant.

Compared to animals, plant exonic circRNAs are less likely to be generated from exon(s) flanked by introns containing repetitive and/or reverse complementary sequences. In rice, Lu, et al. [43] showed that only a very small part of rice circular RNAs contain two flanking sequences overlapping with miniature invertedrepeat transposable elements (MITEs) through searching 500-bp flanking sequences of the backsplice sites. Furthermore, Ye, et al. [21] found that, unlike human exonic circRNAs, most of which are flanked by canonical GT/AG splicing signals, the vast majority of rice circRNAs contain very diverse non-GT/AG splicing signals. To determine whether exon circularization depends on flanking intronic complementary sequences in cucumber, we detected putative complementary sequences by testing 1000-bp flanking sequences of the backsplice sites from circRNAs and only 18 circRNAs were detected to contain flanking complementary sequence longer than 17-bp. Furthermore, we compared the cucumber circRNAs flanking sequences with plant miniature invertedrepeat transposable elements (MITEs) and only 6 circRNAs were found to have two flanking sequences that overlapped with MITEs. To further validate this observation, we expanded the flanking sequences to 2-kb, and, as expected, still a small number of circRNAs (1.5%) have flanking complementary sequences longer than 17-bp, suggesting the biosynthesis of cucumber circRNAs possibly not dependent on flanking complementary sequences (Additional file 3: Table S3). Similar result was also observed in rice. Therefore, in cucumber, other mechanisms may be involved in circRNA generation in addition to pairing-driven circularization.

The responses of plant to salt stress are very complex processes and involve many salt-inducible genes and signaling transduction pathways. In this study, we explored the roles of circRNAs in cucumber in responses to salt stress and found 1966 circRNAs significantly differentially expressed under salt stress compared with CK (Fig. 5. Additional file 8: Table S7, Additional file 9: Table S8). Among these 1966 circRNAs, a large number of them (1934) belong to the root and only 44 belong to the leaf, which may be attributable to: (1) as suggested by previous studies, levels of circular RNAs vary across tissues, species and treatments. For example, circRNAs exhibited specific expression patterns in different tissues of soybean, with 776, 3171 and 2165 being identified from leaves, roots and stems, respectively (Zhao et al. 2017). circRNAs are also treatment-specific. In Arabidopsis, heat stress induced more circRNAs than in normal condition [20]. In barely, circRNAs behaved differently in leaf and seed tissues when comparing the treatments with foliar application of micronutrients [17]. (2) Root is the first organ that respond to salt stress, which senses salt signals to initial reestablishment of cellular ionic homeostasis and osmotic equilibrium under stress conditions [23]. Some stress-responsive genes function primarily in the root. The biological functions of circRNA could be related to the known function of the host linear transcripts [10, 43]. GO enrichment analysis on parent genes of these differentially expressed circRNAs showed that GO terms related to salt stress response including amino acid transport and metabolism, energy production and conversion, and ion transporting were specifically enriched (Fig. 5c. Additional file 8: Table S7) [44]. Thus, a hypothesis was raised that these circRNAs were valuable parts in cucumber in response to salt stress through regulating the expression level of their parent genes.

**Fig. 7:**
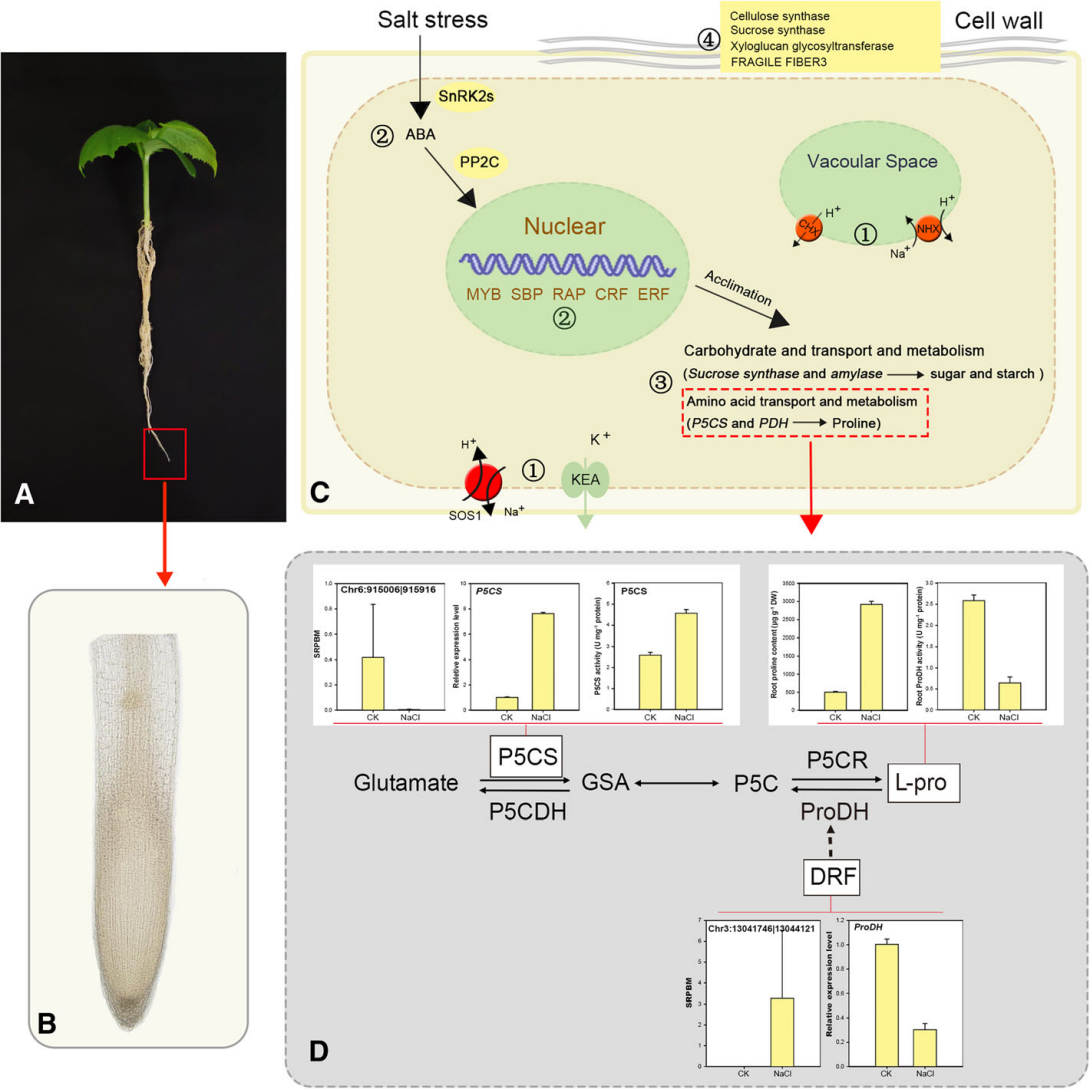
Possible regulatory mechanisms that circRNAs involved in the root of cucumber during salt stress. **a** Cucumber plant. **b** Root system is the first plant organ that senses excess Na^+^, which triggers downstream stress responses. **c** Function annotation of parent gene and mRNA targeted by miRNAs suggested that circRNAs participate in salt-induced physiological and biochemical response including ① Ion homeostasis regulation (e.g. CHX, KEA, NHX), ② activation of abscisic acid (ABA) pathways and osmotic stress signaling (SnRK2s, PP2C, MYB, SBP et al.), ③ biosynthesis and accumulation of compatible osmolytes (proline, sucrose), and ④ physical and chemical alterations of cell wall (cellulose synthase, sucrose synthase, xyloglucan glycosyltransferase; FRAGILE FIBER 3). **d** The schematic summary presents expression levels of circRNAs and proline metabolism-related genes, and P5CS, ProDH activities and proline content with significant differences between treatments. Values are presented as the means of six replicates ± SE. CHX, cation/H^+^ exchanger-like; KEA, K^+^ efflux antiporter-like; NHX, Na+/H+ exchanger-like; SnRK2s, SNF1-related kinases; PP2C, protein phosphatase 2C; P5CS, D1-pyrroline-5-carboxylate synthetase; PDH, proline dehydrogenase

Amino acids have various prominent functions in plants including stress response [77]. Proline (Pro) biosynthesis and degradation are known to undergo alteration in response to diverse environmental stresses. Moreover, proline has been considered to be one of the most commonly occurring osmolytes and scavenger of reactive oxygen species (ROS) under salt stress [77]. In plants, proline is synthesized from glutamate by the action of two enzymes, P5CS and ∆1-pyrroline-5-carboxylate reductase (P5CR). P5CS converts glutamate into pyrroline-5-carboxylate (P5C) and has been proposed as a rate-limiting enzyme involved in the biosynthesis of proline in higher plants. Conversely, proline is oxidized to glutamate via PDH and ∆1-pyrroline-5-carboxylate dehydrogenase (P5CDH) [72]. In this study, circRNA Chr3:13041746|13,044,121 and Chr6:915006|915,916 may act as a negative and positive regulator of their parent genes Csa5G643300 and Csa3G185140 respectively, which were reported to participate in proline biosynthesis and degradation (Fig. 7). The production of circRNAs has been reported to have a negative or positive effect on expression of their parental gene. For example, in rice, Lu, et al. [43] suggested that circRNA and its linear form might act as a negative regulator of its parental gene. Li, et al. [6] proposed that intron-containing circRNAs (EIciRNAs) interact with U1 snRNP and promote transcription of their parental genes. To further explore the relationship between circRNAs and their parent genes, the expression level of 6 random selected circRNAs and their parent mRNAs in different time points were examined. Results suggested that in most cases, the expression patterns of parent mRNAs showed an opposite trend with circRNAs. But there are exceptions, the expression levels of CsCir12 were coincident with the increased expression of their parental genes after 3, 6 and 9 days of treatment (Additional file 15: Figure S3). In barley, weak and negative correlations between cellular levels of circular RNAs and the linear-transcript levels of their parental genes were reported [17]. However, expression profiles of circRNAs in some plant including tea and *Arabidopsis* showed a positive correlation with their parental genes (Tong et al. 2018) [16, 19]. Comprehensive identification and quantification of circRNAs in more plant species are needed to reveal the relationship between the abundances of circRNAs and their corresponding parental genes. The complicate relationship between circRNAs and their parent genes need to be further explored by molecular biology approaches.

## Conclusion

We first demonstrated the existence of abundant circRNAs in cucumber and their possible regulating roles in response to salt stress. Characterization analysis revel two key findings in our work: (1) circRNAs prefer to arise from middle exon(s) of parent genes and specific length of exons are favorited by circularization; (2) Unlike animals, non-GT/AG splicing signals are common in circRNAs and pairing-driven circularization is not the major way to generate circRNAs. Annotation and enrichment analysis on differentially expressed circRNAs reveal that these circRNAs are associated with numerous cucumber coding genes generally functional related to salt stress response, including transcription, signal transcription, cell wall biogenesis, metabolism adaptation (e.g. carbohydrate transport and metabolism, and amino acid transport and metabolism) and ion homeostasis, suggesting circRNAs’ wide involvement of participating in cucumber responding to salt stress (Fig. 7). Our study expands our understanding about the characters of plant circRNAs and the regulating diversity of cucumber transcriptome, which will facilitate the future regulatory mechanism analyses of cucumber circRNA in responding to salt stress.

## Additional files

Additional file 1: **Table S1.** Physical position of unanchored scaffolds in merged ChrUn. (XLSX 17 kb)

Additional file 2: **Table S2.** The mature sequences of reported cucumber miRNAs. (XLSX 87 kb)

Additional file 3: **Table S3.** Primers used for circRNAs sequences validation. (XLSX 13 kb)

Additional file 4: **Table S4.** Detailed information of identified circRNAs. (XLSX 726 kb)

Additional file 5: **Table S5.** circRNAs derived from orthologous genes of cucumber and seven other species. (XLSX 235 kb)

Additional file 6: **Figure S1.** The number of gene pairs in the parent genes of cucumber circRNAs and other species for orthologs. (DOCX 30 kb)

Additional file 7: **Table S6.** circRNAs with complementary or MITE flanking sequences. (XLSX 18 kb)

Additional file 8: **Table S7.** Significantly differentially expressed circRNAs in root. (XLSX 474 kb)

Additional file 9: **Table S8.** Significantly differentially expressed circRNAs in leaf. (XLSX 19 kb)

Additional file 10: **Table S9.** miRNAs sponged by circRNAs. (XLSX 17 kb)

Additional file 11: **Table S11.** miRNAs sponged by significantly differentially expressed circRNAs and their mRNA targets in root. (XLSX 90 kb)

Additional file 12: **Table S12.** miRNAs sponged by significantly differentially expressed circRNAs and their mRNA targets in leaf. (XLSX 39 kb)

Additional file 13: **Table S10.** Evolutionary conserved miRNAs sponged by circRNAs. (XLSX 13 kb)

Additional file 14: **Figure S2.** Chromosome length versus circRNA number. (DOCX 81 kb)

Additional file 15: **Figure S3.** Expression analysis of circRNAs and their parent genes. (DOCX 308 kb)

## Abbreviations

BP: Biological Process
CC: Cellular Component
CHX2-like: Cation/H^+^ exchanger 2-like
circRNA: Circular RNA
FDR: False discovery rate
GO: Gene Ontology
h: Hour
HRCR: Heart-related circRNA
KEA2-like: K^+^ efflux antiporter 2-like protein
KEA6-like: K^+^ efflux antiporter 6-like
KEGG: Kyoto Encyclopedia of Genes and Genomes
KOG/COG: Clusters of Orthologous Groups of proteins
L: Leaf
MF: Molecular Function
MITEs: Miniature invertedrepeat transposable elements
N: Unknown
NCBI: National Center for Biotechnology Information
NHX7-like: Sodium/hydrogen exchanger 7-like protein
Nr: NCBI non-redundant protein sequences
P5CS: D1-pyrroline-5-carboxylate synthetase
PDH: Proline dehydrogenase
PP2C: Protein phosphatase 2C
qRT-PCR: Quality real time PCR
R: Root
SnRKs: SNF1-related kinases
SRPBM: Number of circular reads/number of mapped reads (units in billion)/read length
Swiss-Prot: A manually annotated and reviewed protein sequence database
TFs: Transcription factor
