# Supplementary material for: Identification of cucumber circular RNAs responsive to salt stress
Source: BMC Plant Biol. 2019 Apr 27;19:164. doi: 10.1186/s12870-019-1712-3 (PMC6486992; doi:10.1186/s12870-019-1712-3)
Supplement: Supplementary file 6 — Figure S1. The number of gene pairs in the parent genes of cucumber circRNAs and other species for orthologs. (DOCX 30 kb) [file 12870_2019_1712_MOESM6_ESM.docx]

**
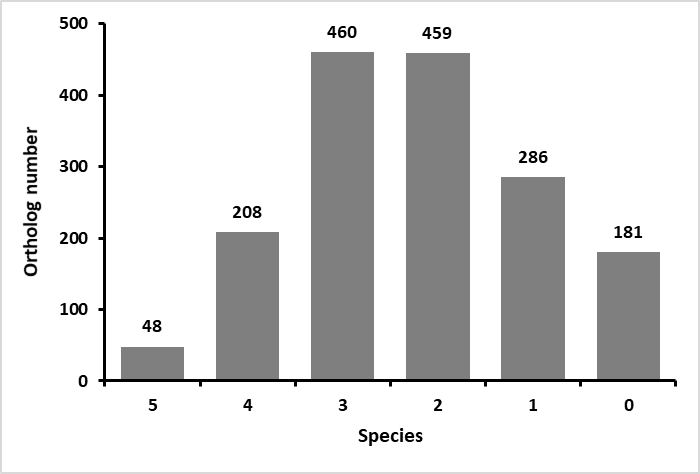
**

**Figure S1.** The number of gene pairs in the parent genes of cucumber circRNAs and other species for orthologs. The 1,642 parent genes that produced exonic circRNAs in cucumber were used to search orthologous sequences in seven species including Arabidopsis, rice, soybean, maize, tomato, potato, and barley in EnsemblPlants database by BioMart. The x-axis represents for number of species that cucumber orthologous genes could be identified.
