# Supplementary material for: Identification of cucumber circular RNAs responsive to salt stress
Source: BMC Plant Biol. 2019 Apr 27;19:164. doi: 10.1186/s12870-019-1712-3 (PMC6486992; doi:10.1186/s12870-019-1712-3)
Supplement: Supplementary file 14 — Figure S2. Chromosome length versus circRNA number. (DOCX 81 kb) [file 12870_2019_1712_MOESM14_ESM.docx]

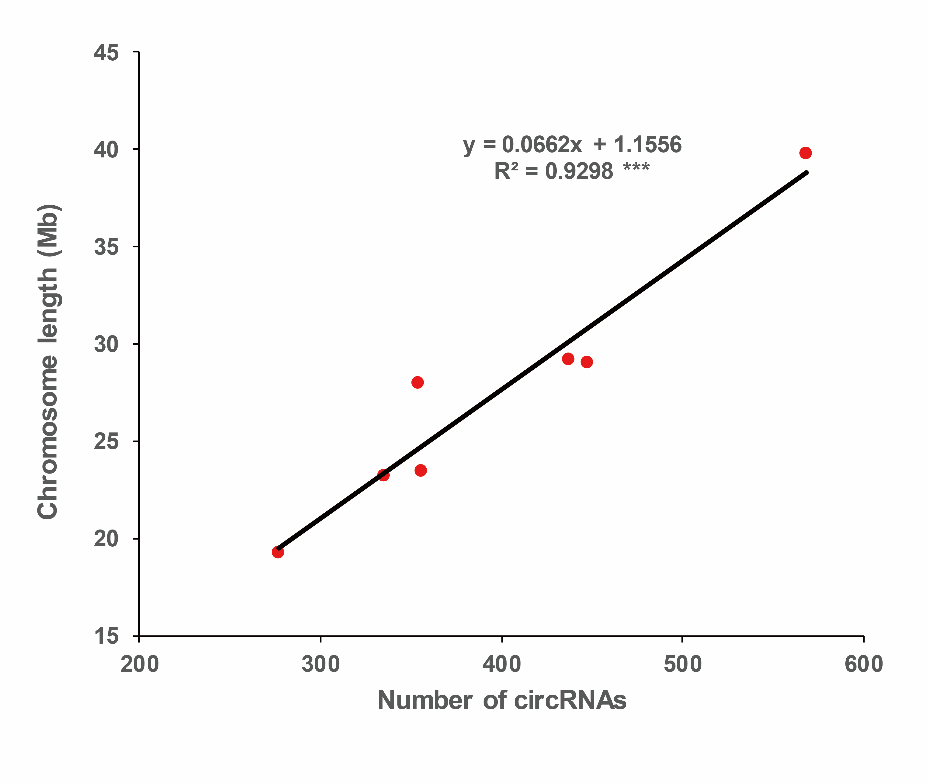


**Figure S2.** The correlation analysis between the chromosome length and the mount of circRNAs in each corresponding chromosome.
