# Supplementary material for: Identification of cucumber circular RNAs responsive to salt stress
Source: BMC Plant Biol. 2019 Apr 27;19:164. doi: 10.1186/s12870-019-1712-3 (PMC6486992; doi:10.1186/s12870-019-1712-3)
Supplement: Supplementary file 15 — Figure S3. Expression analysis of circRNAs and their parent genes. (DOCX 308 kb) [file 12870_2019_1712_MOESM15_ESM.docx]

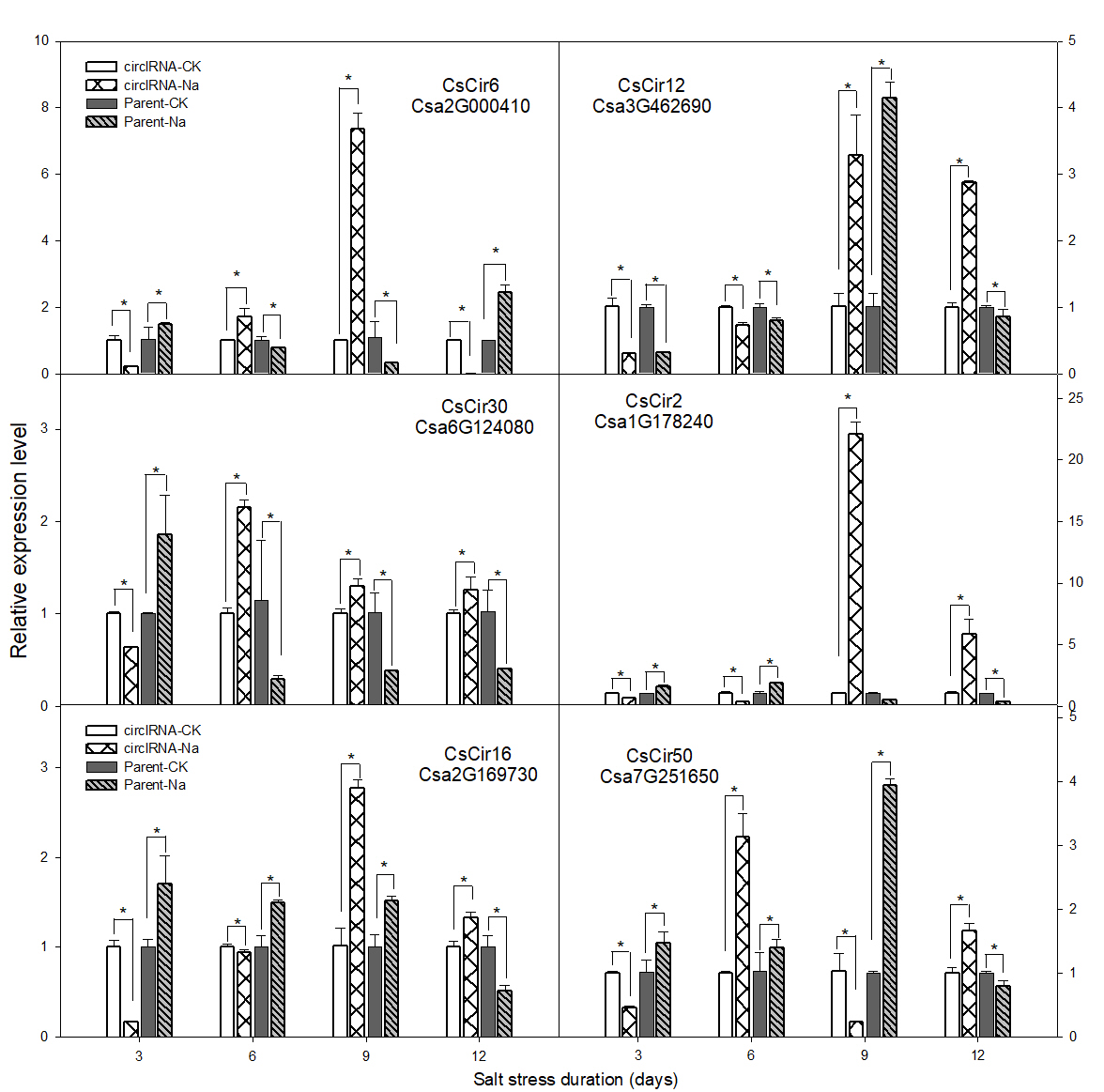


**Figure S3.** Expression analysis of circRNAs and their parent genes. CsCirc2, Csa1G178240 (sodium/hydrogen exchanger); CsCirc6, Csa2G000410 (RecA); CsCirc11, Csa2G169730 (Sec24-like); CsCirc12, Csa3G462690 (Unknown); CsCirc30, Csa6G124080 (Formiminotransferase-cyclodeaminase); CsCirc33, Csa7G251650 (TPR). The relative expression was determined by qRT-PCR. Values are mean ± SD of three replications, and each replication included two technical replications. Asterisk above the columns indicate a significant difference between stress-treated and control plants (*, *p* < 0.05).
